# An improved chaos sparrow search algorithm for UAV path planning

**DOI:** 10.1038/s41598-023-50484-8

**Published:** 2024-01-03

**Authors:** Yong He, Mingran Wang

**Affiliations:** School of Electrical and Information Engineering, Changsha University of Science and Technology, Changsha, 410114 China

**Keywords:** Electrical and electronic engineering, Mechanical engineering

## Abstract

This study suggests an improved chaos sparrow search algorithm to overcome the problems of slow convergence speed and trapping in local optima in UAV 3D complex environment path planning. First, the quality of the initial solutions is improved by using a piecewise chaotic mapping during the population initialization phase. Secondly, a nonlinear dynamic weighting factor is introduced to optimize the update equation of producers, reducing the algorithm's reliance on producer positions and balancing its global and local exploration capabilities. In the meantime, an enhanced sine cosine algorithm optimizes the update equation of the scroungers to broaden the search space and prevent blind searches. Lastly, a dynamic boundary lens imaging reverse learning strategy is applied to prevent the algorithm from getting trapped in local optima. Experiments of UAV path planning on simple and complex maps are conducted. The results show that the proposed algorithm outperforms CSSA, SSA, and PSO algorithms with a respective time improvement of 22.4%, 28.8%, and 46.8% in complex environments and exhibits high convergence accuracy, which validates the proposed algorithm's usefulness and superiority.

## Introduction

With the rapid development of drone technology, drones have found further applications in various industries. The mission scenarios for drones have also evolved from open spaces to spaces with irregularly distributed obstacles^1^. The topic of UAV route planning^2^ can be treated as a constrained multi-objective optimization problem, with the main goal of autonomously searching for the optimal or suboptimal trajectory from the starting point to the target point based on mission objectives and flying limitations. In complex environments, UAV path planning has higher requirements, and traditional optimization methods such as A^*^^3^ and RRT^4^ are no longer able to simultaneously satisfy the requirements for both time efficiency and accuracy.

Taking inspiration from biological behaviors in nature, various swarm intelligence optimization algorithms have been introduced and applied to the problem of UAV path planning. These algorithms include genetic algorithms^5^, particle swarm optimization^6^, ant colony optimization^7^, and grey wolf optimization^8^, Beetle Swarm Optimization Algorithm^9^, Whale Optimization Algorithm^10^, Manta ray foraging optimization^11^. These algorithms can employ different search strategies for multiple iterations to quickly find effective paths. However, how to prevent the algorithms from getting trapped in local optima remains one of the key challenges that most intelligent algorithms need to address^12^.

The Sparrow Search Algorithm (SSA)^13^, invented by Xue in 2020, is a novel heuristic search algorithm that mimics the path selection, search, and flight processes of sparrows during foraging behavior. By introducing certain random factors, the algorithm aims to enhance the breadth and diversity of the search process, thus preventing it from getting trapped in local optima. Liu et al^14^. proposed a novel improved Sparrow Search Algorithm (CSSSA) that utilizes Cubic chaos mapping to enhance the randomness of the initial population. The algorithm employs adaptive inertia weight to improve its exploration capability and convergence speed. Additionally, the Cauchy-Gaussian mutation strategy is applied to help the algorithm escape local optima. Finally, the improved algorithm is validated in complex three-dimensional path planning for drones to demonstrate its superior performance. Song et al^15^. used the Circle chaos mapping to optimize the initial population. They combined the Tunicate Swarm Algorithm with an adaptive step size factor to enhance the algorithm’s global exploration capability and convergence speed. Furthermore, the algorithm incorporates a simulated annealing mechanism to efficiently escape local optima. The performance of the improved algorithm is demonstrated through experiments on eight test functions, showcasing its excellent optimization ability. Yu et al^16^. employed a Pareto set initialization strategy, utilized adaptive dynamic weights to balance global and local search capabilities, strengthened the local exploration capability with Gaussian mutation, and used the Tent chaotic sequence to avoid being trapped in local optima. Yan et al^17^. utilize the Latin Hypercube Sampling in the initialization process to make the population more evenly distributed. They introduce an improved iterative local search to make the algorithm more flexible in its search capability. Finally, they use a coordinate-wise lens imaging inverse learning method to help the algorithm escape local optima and improve convergence speed. Experiments on 12 test functions demonstrate that the improved algorithm has significantly improved optimization accuracy and stability. Ouyang et al^18^. propose a Reflected Sparrow Search Algorithm, which updates the discoverer’s position through reflected inverse learning to expand the search space. They add a crazy operator to the followers to refine the search. Finally, they combine the simulated annealing algorithm to refine the solution of each iteration and find the optimal solution. The improved algorithm is applied to drone path planning, and the results show that the algorithm has the lowest cost. Jia et al^19^. Optimize the initial population using the Circle chaos mapping. They expand the range of the discoverer update equation to Circle and the range of the sentinel to Chebyshev. They apply chaos mapping to clamp individuals outside the search range. Experiments on various representative CEC functions demonstrate that the improved algorithm has significant advantages in population diversity and convergence speed.

While the aforementioned research has achieved faster convergence by balancing the algorithm's global and local exploration capabilities, they fall short in terms of search accuracy and do not sufficiently explore uncharted territory in the search space. To address the aforementioned issues, this study proposes four improvement strategies: (1) Utilizing the nonlinearity and exploratory characteristics of chaotic mappings, the population initialization is improved using Piecewise chaotic mapping to enhance population dispersion and improve global search capability. (2) A nonlinear dynamic weighting factor is employed to optimize the discoverer update equation, balancing the algorithm's local and global exploration capabilities and enabling fast convergence in later iterations to accelerate convergence speed. (3) By integrating the improved Sine Cosine Algorithm into the predator update equation, the search range is expanded, avoiding blind searching and facilitating information exchange among individuals in the population. (4) To prevent the algorithm from getting trapped in local optima, a lens imaging inverse learning approach is used to update the population positions. By controlling the learning boundaries, the updated population is prevented from crossing the boundaries of the search space, thereby increasing the diversity of the algorithm's optimization solutions. Finally, the proposed algorithm is applied to three-dimensional path planning for unmanned aerial vehicles to verify its feasibility.

## Algorithm foundation

The Sparrow Search Algorithm is a novel swarm intelligence optimization algorithm that simulates the foraging and predator evasion behavior of sparrows. In SSA, there are two main roles: producer and scrounger, and a reconnaissance and warning strategy is incorporated. The top 10–20% of individuals with high energy or fitness values in the population act as producers to search for food and share the discovered food locations with other individuals, while scroungers acquire food based on this information. During the foraging process, the energy of sparrow individuals changes, allowing the roles of producers and scroungers to switch. Additionally, 10–20% of individuals in the population can perceive danger signals. When encountering predators, they emit signals to alert the population to abandon foraging and move to safer areas. Ultimately, through iterative transformations of sparrow individuals' positions, the optimal food source is discovered.

In the SSA algorithm, producer, scroungers, and watchdog, each sparrow updates its position according to specific update rules during the search process. The following are the updated rules:

1 $$X_{i,j}^{t + 1} = \left\{ \begin{gathered} X_{i,j}^{t} \cdot \exp \left( {\frac{ - i}{{\alpha \cdot iter_{\max } }}} \right)\quad {\text{if R}}_{2} < ST \hfill \\ X_{i,j}^{t} + Q \cdot L\,\,\,\,\,\quad \quad \quad \quad {\text{if R}}_{2} \ge ST \hfill \\ \end{gathered} \right.$$

where ${X}_{i,j}^{t}$ represents the value of the i sparrow in the j dimension at the t iteration; $t$ represents the current number of iterations; and $M$ represents the maximum number of iterations. $\alpha$ is a number chosen at random between (0,1); ${R}_{2}$ and $ST$ represent alarm value and safety threshold, and their values are [0,1] and [0.5,1.0], respectively. $Q$ has a normal distribution and is generated at random. $L$ is a 1 by d matrix with all 1's. When ${R}_{2}<ST$, it means that there are no natural enemies around to continue the search. When ${R}_{2}>ST$, it indicates that there are natural enemies at the current location, and the watcher will send a signal to warn other sparrows to move to the safe area.

2 $$X_{i,j}^{t + 1} = \left\{ \begin{gathered} Q \cdot \exp \left( {\frac{{X_{worst}^{t} - X_{i,j}^{t} }}{{i^{2} }}} \right)\quad \quad \;\;\;\;{\text{if i > n/2}} \hfill \\ {\text{X}}_{P}^{t + 1} { + }\left| {X_{i,j}^{t} - X_{p}^{t + 1} } \right| \cdot D^{ + } \cdot L\quad \quad {\text{if i}} \le {\text{n/2}} \hfill \\ \end{gathered} \right.$$

where ${X}_{p}$ denotes the current best position of the discoverers; ${X}_{worst}$ represents the current global worst position; $D$ is a 1 × d matrix with random elements of either 1 or − 1, and ${D}^{+}={D}^{T}\cdot {\left(D{D}^{T}\right)}^{-1}$; $n$ denotes the number of sparrows in the population; when i > n/2, it indicates that the $i - {\text{th}}$ predator is in a hungry state and needs to fly to another location to search for food.

3 $$X_{i,j}^{t + 1} = \left\{ \begin{gathered} X_{best}^{t} + \beta \cdot \left| {X_{i,j}^{t} - X_{best}^{t} } \right|\quad \quad {\text{if f}}_{i} > {\text{f}}_{g} \hfill \\ X_{i,j}^{t} + k \cdot \left( {\frac{{\left| {X_{i,j}^{t} - X_{best}^{t} } \right|}}{{\left( {f_{i} - f_{w} } \right) + \varepsilon }}} \right)\quad {\text{if f}}_{i} = {\text{f}}_{g} \hfill \\ \end{gathered} \right.$$

where ${X}_{best}$ denotes the current top spot on the worldwide ranking; ${f}_{i}$ represents the fitness value of the current sparrow, ${f}_{g}$ and ${f}_{w}$ represent the current global best and worst fitness values, and $\varepsilon$ is a small constant to prevent division by zero. $\beta$ is a random number with a normal distribution with mean 0 and variance 1, used to control the step size; $k$ is a random number in the range [-1,1], used to control the direction of the sparrow movement. When ${f}_{i}>{f}_{g}$, it indicates that the individual is at the population’s edge and is more likely to be preyed upon by predators; when ${f}_{i}={f}_{g}$, it implies that sparrows have detected danger and need to move closer to other individuals to avoid being preyed upon.

## Algorithm improvements

### Chaos mapping

In recent years, many researchers have incorporated chaotic sequences^20^ into swarm intelligence optimization algorithms. Chaotic sequences possess characteristics such as nonlinearity, ergodicity, and randomness. The initial population with good distribution is the basis of the swarm intelligence optimization algorithm. By adding chaotic mapping during population initialization, individuals can be more evenly distributed in the search space, which will improve population diversity and global search ability.

Commonly used chaotic mappings include the Logistic map^21^, the Tent map^22^, and the Piecewise map^23^. In this paper, we utilize the Piecewise chaotic mapping to improve the population initialization process. The Piecewise map, also known as the PWLCM map (Piecewise Linear Chaotic Map), is a typical piecewise linear mapping that applies different transformation equations to the initial values within different intervals. The PWLCM mapping equation is represented as follows:

4 $$x(t + 1) = \left\{ {\begin{array}{*{20}c} {\frac{x(t)}{p},} & {0 \le x(t) < p} \\ {\frac{x(t) - p}{{0.5 - p}},} & {p \le x(t) < 0.5} \\ {\frac{1 - p - x(t)}{{0.5 - p}},} & {0.5 \le x(t) < 1 - p} \\ {\frac{1 - x(t)}{p},} & {1 - p \le x(t) < 1} \\ \end{array} } \right.$$

where $p$ is the parameter that controls the four segments to ensure non-overlapping intervals. In this paper, we set *p* = 0.4.

**Figure 1:**
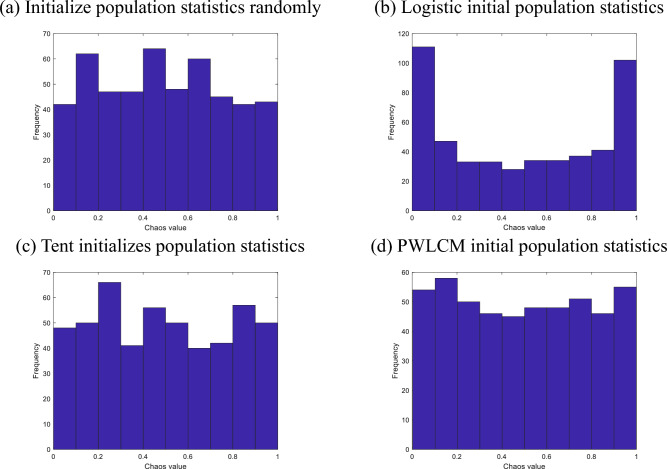
Initial population statistics.

Figure 1a–d shows the initial population distribution statistics for random, logistic, tent, and PWLCM, respectively. As can be seen, the initial population distribution is more uniform after PWLCM mapping, and the sparrow population can search a wider space, which significantly reduces the issue that the original algorithm is prone to settle for the local optimal solution.

#### Nonlinear Dynamic Weight Factor

**Figure 2:**
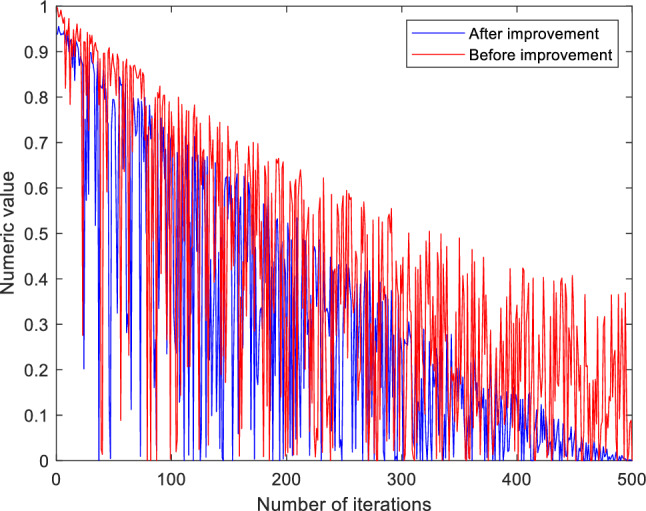
Finder search policy.

It can be seen from Fig. 2 that the producer relies too much on the update method of its position in the early iteration, resulting in insufficient search ability and slow convergence. Later iterations are easily limited by the local best solution and cannot be further optimized. As a result, the dynamic weight factor optimization producer update equation is used to balance global and local exploration capabilities while increasing the algorithm's late convergence speed. The weight coefficients are expressed as follows:

5 $$\omega_{d} = \frac{{e^{4(1 - t/M)} - 1}}{{e^{4(1 - t/M)} + 1}}$$

When the algorithm was in its early stages, the dependence of the producer on the previous generation’s position should be avoided, and a larger space should be searched to improve the global development ability. In the later stage, convergence should be attained as quickly as feasible to the global optimal to speed up convergence. Therefore, the Eq. (5) is brought in (1), and the position of the producer after the improvement is updated as follows:

6 $$X_{x,j}^{t + 1} = \left\{ {\begin{array}{*{20}l} {X_{x,j}^{t} \cdot \omega_{d} \cdot \exp \left( {\frac{ - i}{{\alpha \cdot M}}} \right)} \hfill & {R_{2} < ST} \hfill \\ {X_{x,j}^{t} + Q} \hfill & {R_{2} \ge ST} \hfill \\ \end{array} } \right.$$

#### Improved sine cosine algorithm

The scroungers of SSA search for the location of the scroungers immediately after the producer, and the dynamic parameters are few, which makes it easy to reduce the search range, resulting in the blindness of the search. Therefore, the sine cosine algorithm^24,25^ is adopted in the following stage, and its characteristics are used to dynamically change the orientation of individuals, broaden the exploration space, reduce search blindness, speed the sharing of information among individuals in the population, and use the ${r}_{1}$ of linear decline strategy to balance global and local search. Update the equation for the scrounger position using the modified sine cosine strategy:

7 $$X_{i}^{t} = \left\{ {\begin{array}{*{20}l} {X_{i}^{t} + r_{1} \cdot \sin (r_{2} ) \cdot \left| {r_{3} \cdot X_{p}^{t} - X_{i}^{t} } \right|} \hfill & {r_{4} \le 0.5} \hfill \\ {X_{i}^{t} + r_{1} \cdot \cos (r_{2} ) \cdot \left| {r_{3} \cdot X_{p}^{t} - X_{i}^{t} } \right|} \hfill & {r_{4} > 0.5} \hfill \\ \end{array} } \right.$$

8 $$r_{1} = 2 \cdot \exp \left( { - 30 \cdot \left( {t/M} \right)^{5} } \right)$$

where ${r}_{1}$ denotes the following position's movement direction. The larger the number of iterations, the smaller the ${r}_{1}$, and the smaller the search area of the sparrow; ${r}_{2}$ is a random value between [0,2], determining how far an individual moves towards or away from the center; ${r}_{3}$ and ${r}_{4}$ are randomized numbers within [0,2] and [0,1], respectively.

#### Imaging lens reverse learning

**Figure 3:**
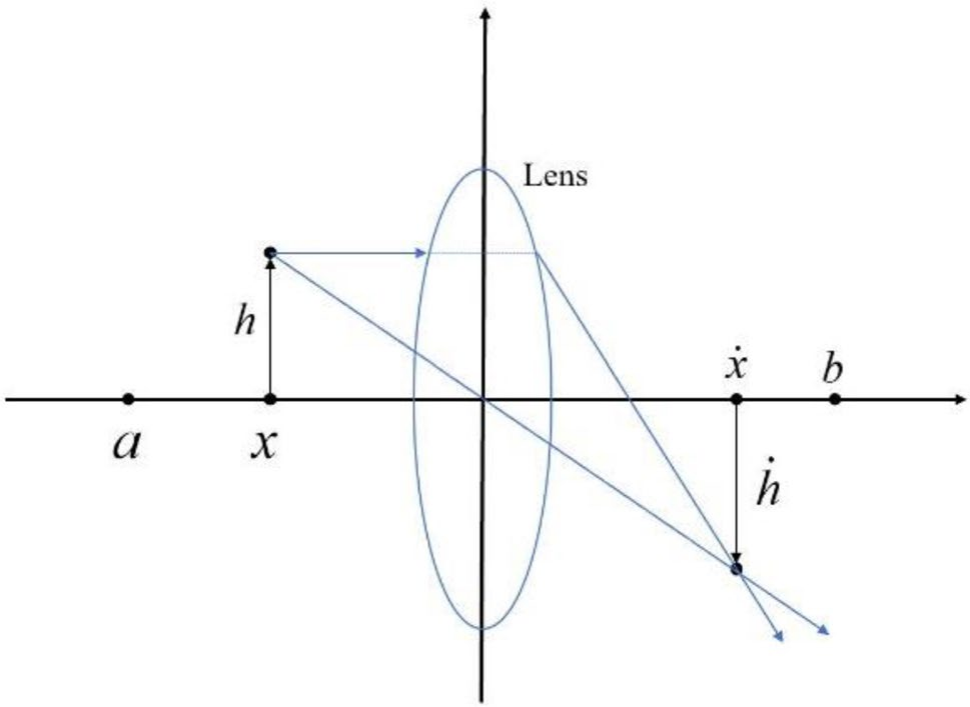
Principle diagram of imaging lens reverse learning.

According to Eqs. (1) and (2), both the producer and the scroungers want to search the solution space for the best solution. However, when both producers and scroungers converge to a localized optimum, the algorithm is prone to becoming captured. Although Eq. (3) can help the algorithm escape the local optima, it is easier to fall into the local optima when applied to complex high-dimensional problems of UAV path planning. Therefore, a reverse learning strategy ^26,27^ is introduced in the algorithm. In general, the reverse learning strategy finds the best answer in a fixed space, which might lead to local optima and monotony. The imaging lens reverse learning^28,29^ provides better optimization capability compared to the general reverse learning strategy, enabling the algorithm to escape local optima and continuously converge to the optimum solution in the solution space. The principle of imaging lens reverse learning is illustrated in Fig. 3.

The process of transforming a regular solution into a reverse solution can be viewed as an imaging process with a lens. Assuming a particle with an altitude of $h$ at position $x$ in the one-dimensional spatial interval [a, b], a lens is positioned at the base position $o$ (where the base position is defined as $\left(a+b\right)/2$), forming an image with a height of $\dot{h}$ at position $\dot{x}$. According to the principle of imaging:

9 $$\frac{(a + b)/2 - x}{{\dot{x} - (a + b)/2}} = \frac{h}{{\dot{h}}} = k$$

By transforming Eq. (9), we can obtain the calculation equation for the reverse point $\dot{x}$:

10 $$\dot{x} = (a + b)/2 + (a + b)/2k - x/k$$

where $k$ is a scaling factor used to represent the correspondence between the object and the image. When k = 1, Eq. (11) can be simplified as:

11 $$\dot{x} = a + b - x$$

At this point, we have a generic equation for reverse learning, and it is clear that lens image reverse learning is a subset of general reverse learning. The reverse solution in general reverse learning is deterministic, while lens imaging reverse learning can obtain diverse reverse solutions by adjusting the value of $k$, thereby improving algorithm optimization accuracy. This article proposes the use of a linearly increasing strategy to adjust the parameter $k$, which is:

12 $$k = d + t/M$$

where $d$ is a tiny constant and $M$ is the number of iterations to the maximum. When $k$ is small in the early stage, the imaging is larger. When $k$ increases later in the stage, the imaging becomes slightly smaller, which helps with convergence. To avoid excessive imaging in the early stage, this paper chooses $d=0.2$.

Expanding Eq. (11) to a D-dimensional optimization problem, we obtain the equation for lens imaging reverse learning:

13 $$\dot{x}_{j} = (a_{j} + b_{j} )/2 + (a_{j} + b_{j} )/2k - x_{j} /k$$

where ${a}_{j}$ and ${b}_{j}$ are the upper and lower bounds of the j-th dimension, respectively. ${x}_{j}$ represents the current individual's component in the j-th dimension. Furthermore, dynamic boundaries are used in this paper.

14 $$\begin{gathered} a_{j} = low(x_{j} ) \hfill \\ b_{j} = up(x_{j} ) \hfill \\ \end{gathered}$$

where $low\left({x}_{j}\right)$ and $up\left({x}_{j}\right)$ are the lower and upper boundaries of the current population in dimension j, respectively. Because ${a}_{j}$ and ${b}_{j}$ are the top and lowest edges of the j-th dimension, imaging that goes beyond the boundaries $\left[{a}_{j},{b}_{j}\right]$ may not surpass the solution space. Therefore, when $k$ is small, imaging beyond the current j-th dimension boundary is advantageous for expanding exploration space, reducing the likelihood of premature stagnation, and also helping to escape local optima. Finally, a greedy method is used to keep individuals with higher fitness levels.

15 $$x_{i,j}^{t + 1} = \left\{ {\begin{array}{*{20}l} {\frac{{a_{j}^{t} + b_{j}^{t} }}{2} + \frac{{a_{j}^{t} + b_{j}^{t} }}{2k} - \frac{{x_{i,j}^{t + 1} }}{k},} \hfill & {f(\dot{x}) < f(x)} \hfill \\ {x_{i,j}^{t + 1} ,} \hfill & {f\left( {\dot{x}} \right) \ge f(x) \, } \hfill \\ \end{array} } \right.$$

#### Algorithmic processes

**Figure 4:**
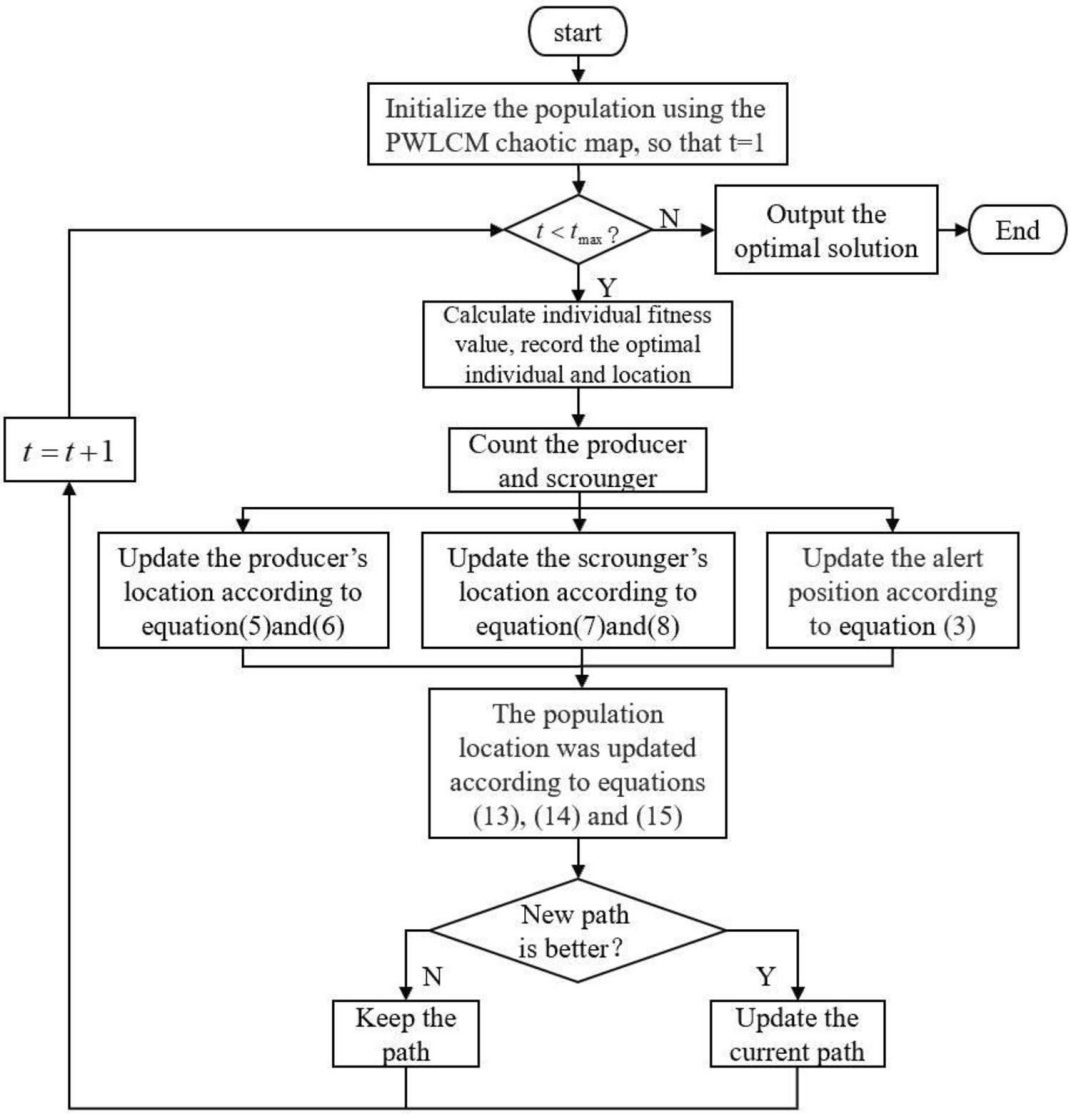
Algorithm flowchart.

An improved chaos sparrow search algorithm (ICSSA) is proposed in this work. Firstly, the initial population is improved using a Piecewise chaotic mapping to achieve a more uniform distribution and enhance the algorithm’s global exploration capability. Then, a nonlinear dynamic weight balancing technique is used to balance the local and global exploration abilities, boosting convergence speed. Following that, the modified sine–cosine algorithm is used to optimize the scrounger’s update equation, preventing blind searching for and increasing the predator’s exploration capability. Finally, lens imaging reverse learning is utilized to prevent getting trapped in local optima. Implementing these strategies increases the algorithm's optimization flexibility, increases population diversity, strengthens the algorithm's ability to flee local optimal situations, balances the algorithm's global and local exploitation skills, and facilitates the discovery of feasible solutions. The algorithm flowchart is shown in Fig. 4.

## Algorithm testing

### CEC2022

To verify the effectiveness and superiority of the proposed algorithm, it was validated using CEC2022 and compared with SSA, CSSA^20^, MSSSA^30^, SCHHO^31^, and MWOA^32^. To ensure fair comparisons, the maximum evaluation times were set to 10,000 times the dimension, and the population size was set to 50. In this study, tests were conducted for both 10 and 20 dimensions. The algorithm was independently run 30 times, and the average (Ave), standard (Std), and Best were recorded to evaluate the optimization ability of the algorithm. The test results are shown in Tables 1 and 2. How the algorithm iterates to the optimal solution is also an important criterion for judging the algorithm's performance. The purpose of the improved algorithm is to solve the optimal or suboptimal solution in the least amount of time. Figure 4 shows the average convergence curves for each algorithm when tested in 10 dimensions. Additionally, to illustrate the differences from other algorithms more intuitively, the Wilcoxon rank-sum test was used with a significance level of 5%. When ICSSA outperformed other algorithms, it was represented by “ + ”. When it was comparable to other algorithms, it was represented by “ = ” and when it performed worse than other algorithms, it was represented by “-”. All experiments in this study were conducted on the Windows 11 platform with Intel Core i5-12500H CPU@2.5GHz using MATLAB 2019b software for simulation experiments.

**Table 1:** Test results for each algorithm in CEC2022 with the dimension of 10.

Function	Index	SSA	CSSA	MSSSA	SCHHO	MWOA	ICSSA
F1	Best	3.5391E + 02	3.0233E + 02	3.0701E + 02	**3.000E + 02**	2.2208E + 03	**3.0000E + 02**
	Ave	1.9025E + 03	3.1526E + 02	4.0222E + 02	3.0002E + 02	3.0746E + 02	**3.0000E + 02**
	Std	1.1618E + 03	3.7695E + 01	1.4615E + 02	2.2636E-07	1.5329E + 01	**1.6900E-09**
	P	+	+	+	+	+	
F2	Best	4.0046E + 02	4.2050E + 02	4.0051E + 02	**4.0000E + 02**	4.0006E + 02	**4.0000E + 02**
	Ave	4.7526E + 02	5.5339E + 02	4.2666E + 02	4.0909E + 02	4.0916E + 02	**4.0405E + 02**
	Std	2.4508E + 01	2.8069E + 01	2.5006E + 01	1.4953E + 01	1.4929E + 01	**3.6746E + 00**
	P	+	+	+	=	=	
F3	Best	6.2022E + 02	6.0400E + 02	**6.0000E + 02**	6.0046E + 02	6.0258E + 02	**6.0000E + 02**
	Ave	6.2067E + 02	6.0965E + 02	6.0006E + 02	6.0004E + 02	6.0512E + 02	**6.0000E + 02**
	Std	6.8663E + 00	6.7681E + 00	**2.6881E-02**	1.2909E + 00	1.2907E + 00	2.0336E + 00
	P	+	+	**-**	-	+	
F4	Best	8.0719E + 02	8.2291E + 02	8.2400E + 02	8.0396E + 02	8.1384E + 02	**8.0246E + 02**
	Ave	8.4436E + 02	8.2568E + 02	8.2580E + 02	**8.1327E + 02**	8.2918E + 02	8.2219E + 02
	Std	8.1246E + 00	1.1221E + 01	8.1103E + 00	7.6845E + 00	2.2663E + 01	**6.1324E + 00**
	P	+	=	=	-	=	
F5	Best	9.0436E + 02	9.2175E + 02	**8.4127E + 02**	9.0154E + 02	1.0552E + 03	9.0000E + 02
	Ave	9.8932E + 02	**9.8705E + 01**	9.0288E + 02	9.0569E + 02	1.2646E + 03	9.0167E + 02
	Std	8.7109E + 01	1.0170E + 02	7.7626E + 00	1.6549E + 01	2.6155E + 02	**3.5486E + 00**
	P	+	+	=	=	+	
F6	Best	1.9235E + 05	2.0481E + 03	2.8622E + 03	**2.8000E + 02**	1.8944E + 03	1.8239E + 03
	Ave	2.0454E + 06	5.1022E + 03	1.9864E + 04	**2.0543E + 03**	3.6325E + 03	2.6126E + 03
	Std	2.7291E + 06	2.5983E + 03	7.0000E + 03	2.0931E + 03	**1.1107E + 03**	1.2223E + 03
	P	+	+	+	-	**-**	
F7	Best	2.0096E + 03	2.0007E + 03	2.0051E + 03	2.0106E + 03	2.0097E + 03	**2.0000E + 03**
	Ave	2.0649E + 03	2.0264E + 03	2.0276E + 03	2.0217E + 03	2.0406E + 03	**2.0200E + 03**
	Std	8.8394E + 00	1.3883E + 01	1.0961E + 01	**4.3675E + 00**	2.1495E + 01	1.0355E + 01
	P	+	+	+	** = **	+	
F8	Best	2.2082E + 03	2.2049E + 03	2.2345E + 03	2.2136E + 03	**2.1348E + 03**	2.2000E + 03
	Ave	2.2325E + 03	2.2256E + 03	2.2312E + 03	2.2256E + 03	2.2254E + 03	**2.2200E + 03**
	Std	3.3426E + 00	3.2491E + 00	7.1427E + 00	6.3666E + 00	7.0501E + 00	**3.1063E + 00**
	P	+	+	+	=	+	
F9	Best	2.5303E + 03	2.5300E + 03	2.5362E + 03	2.5342E + 03	**2.5128E + 03**	2.5317E + 03
	Ave	2.5830E + 03	2.5367E + 03	2.5268E + 03	2.5318E + 03	2.5315E + 03	**2.5300E + 03**
	Std	2.1080E + 01	3.2619E + 01	5.0189E + 00	6.6041E + 00	1.1291E + 00	**1.0422E-13**
	P	+	+	+	=	+	
F10	Best	2.5079E + 03	2.5086E + 03	2.5006E + 03	2.5165E + 03	**2.4269E + 03**	2.5000E + 03
	Ave	2.5227E + 03	2.5795E + 03	2.5019E + 03	2.5335E + 03	2.5473E + 03	**2.5003E + 03**
	Std	2.4453E + 02	6.6126E + 01	2.1651E-01	5.2541E + 01	6.5325E + 01	**4.9157E-02**
	P	+	+	+	+	+	
F11	Best	2.6507E + 03	2.6011E + 03	2.6049E + 03	2.6439E + 03	2.6026E + 03	**2.6000E + 03**
	Ave	2.7848E + 03	2.7065E + 03	2.7268E + 03	2.7140E + 03	2.683E + 03	**2.6342E + 03**
	Std	**1.7119E + 01**	2.0618E + 02	1.1085E + 02	1.1067E + 02	1.3825E + 02	7.8519E + 01
	P	** + **	+	+	=	+	
F12	Best	2.8637E + 03	2.8774E + 03	**2.8406E + 03**	2.8532E + 03	2.8555E + 03	2.8551E + 03
	Ave	2.8653E + 03	2.8731E + 03	2.8693E + 03	**2.8600E + 03**	2.8721E + 03	**2.8600E + 03**
	Std	1.7057E + 00	1.9837E + 01	1.6122E + 01	2.0135E + 00	3.2462E + 00	**1.2612E + 00**
	P	+	+	+	=	=	

Significant values are in [bold].

**Table 2:** Test results for each algorithm in CEC2022 with the dimension of 20.

Function	Index	SSA	CSSA	MSSSA	SCHHO	MWOA	ICSSA
F1	Best	2.0437E + 04	2.5124E + 03	7.9681E + 02	2.0136E + 04	7.5518E + 03	**3.0000E + 02**
	Ave	3.8724E + 04	6.7096E + 03	8.7258E + 03	4.0349E + 04	1.8733E + 04	**3.0121E + 02**
	Std	8.2759E + 03	3.1727E + 03	4.3290E + 03	9.8107E + 03	1.3919E + 04	**5.6131E-01**
	P	+	+	+	+	+	
F2	Best	1.1705E + 03	4.6384E + 02	4.4502E + 02	4.3449E + 02	4.7443E + 02	**4.0676E + 02**
	Ave	2.3914E + 03	5.9836E + 02	4.9873E + 02	5.0345E + 02	5.3118E + 02	**4.5237E + 02**
	Std	6.1036E + 02	8.0926E + 01	4.4828E + 01	4.1448E + 01	5.1105E + 01	**1.3910E + 01**
	P	+	+	+	+	+	
F3	Best	6.4108E + 02	6.2608E + 02	6.0121E + 02	6.2118E + 02	6.4623E + 02	**6.0000E + 02**
	Ave	6.6904E + 02	6.4861E + 02	6.0324E + 02	6.3647E + 02	6.6508E + 02	**6.0000E + 02**
	Std	9.4777E + 00	9.0230E + 00	1.9457E + 00	7.8113E + 00	1.2742E + 01	**1.1823E-01**
	P	+	+	+	+	+	
F4	Best	9.3106E + 02	8.4937E + 02	8.2061E + 02	8.4116E + 02	8.5913E + 02	**8.0615E + 02**
	Ave	9.6151E + 02	8.7861E + 02	8.4707E + 02	8.7205E + 02	9.0968E + 02	**8.1904E + 02**
	Std	1.5619E + 01	1.3527E + 01	2.1620E + 01	1.7199E + 01	3.7742E + 01	**7.6542E + 00**
	P	+	+	+	+	+	
F5	Best	2.2808E + 03	1.5287E + 03	9.0865E + 02	1.4631E + 03	2.28E + 03	**9.0000E + 02**
	Ave	3.1003E + 03	2.1421E + 03	9.8759E + 02	2.1110E + 03	3.72E + 03	**9.0073E + 02**
	Std	4.1343E + 02	2.9101E + 02	3.3312E + 02	3.7569E + 02	9.85E + 02	**4.0223E-03**
	P	+	+	+	+	+	
F6	Best	1.9361E + 03	2.4096E + 03	2.8372E + 03	1.9343E + 04	4.5913E + 03	**1.9108E + 03**
	Ave	3.7145E + 03	8.2503E + 04	4.2349E + 05	9.2621E + 04	1.1905E + 05	**3.6026E + 03**
	Std	2.5491E + 03	**2.4926E + 02**	1.2463E + 04	6.0936E + 04	1.2222E + 05	2.1004E + 03
	P	+	** + **	+	+	+	
F7	Best	2.1252E + 03	2.0514E + 03	2.0352E + 03	2.0613E + 03	2.0943E + 03	**2.0241E + 03**
	Ave	2.1809E + 03	2.0930E + 03	**2.0726E + 03**	2.1141E + 03	2.1908E + 03	2.0824E + 03
	Std	3.0931E + 01	**2.5463E + 01**	4.1529E + 01	2.6126E + 01	5.2156E + 01	3.2218E + 01
	P	+	** + **	+	+	=	
F8	Best	2.2410E + 03	2.2329E + 03	2.2233E + 03	2.2367E + 03	2.2313E + 03	**2.2200E + 03**
	Ave	2.3220E + 03	2.2508E + 03	2.2536E + 03	2.2542E + 03	2.3167E + 03	**2.2320E + 03**
	Std	7.0118E + 01	4.3192E + 01	5.0253E + 01	4.9490E + 01	7.6327E + 01	**2.1897E + 01**
	P	+	+	+	+	+	
F9	Best	2.7200E + 03	2.4820E + 03	**2.4800E + 03**	2.4917E + 03	2.4930E + 03	**2.4800E + 03**
	Ave	3.1902E + 03	2.5472E + 03	2.5125E + 03	2.5444E + 03	2.5301E + 03	**2.4846E + 03**
	Std	2.7167E + 02	2.9347E + 01	1.9892E + 01	4.1527E + 01	2.8839E + 01	**5.3971E + 00**
	P	+	+	=	+	+	
F10	Best	2.5918E + 03	2.5016E + 03	**2.5000E + 03**	2.5004E + 03	2.5049E + 03	**2.5000E + 03**
	Ave	5.9037E + 03	3.3939E + 03	3.3632E + 03	2.8926E + 03	4.4650E + 03	**2.5875E + 03**
	Std	1.1729E + 03	1.0129E + 03	6.2427E + 02	8.2832E + 02	1.1822E + 03	**3.7729E + 02**
	P	+	+	=	+	+	
F11	Best	6.3363E + 03	3.4183E + 03	2.9521E + 03	2.6904E + 03	2.7599E + 03	**2.6000E + 03**
	Ave	8.2721E + 03	4.3237E + 03	3.3811E + 03	3.0526E + 03	3.1452E + 03	**2.9235E + 03**
	Std	6.9701E + 02	6.6560E + 02	2.1748E + 02	**1.4772E + 02**	2.0316E + 02	1.5157E + 02
	P	+	+	+	** = **	+	
F12	Best	3.2908E + 03	2.9513E + 03	**2.9409E + 03**	2.9641E + 03	2.9644E + 03	2.9530E + 03
	Ave	3.8338E + 03	3.0271E + 03	**2.9744E + 03**	3.0151E + 03	3.0627E + 03	2.9729E + 03
	Std	2.9351E + 02	6.4524E + 01	**2.1853E + 01**	2.4915E + 01	1.0126E + 02	3.5460E + 01
	P	+	+	**-**	+	+	

Significant values are in [bold].

**Figure 5:**
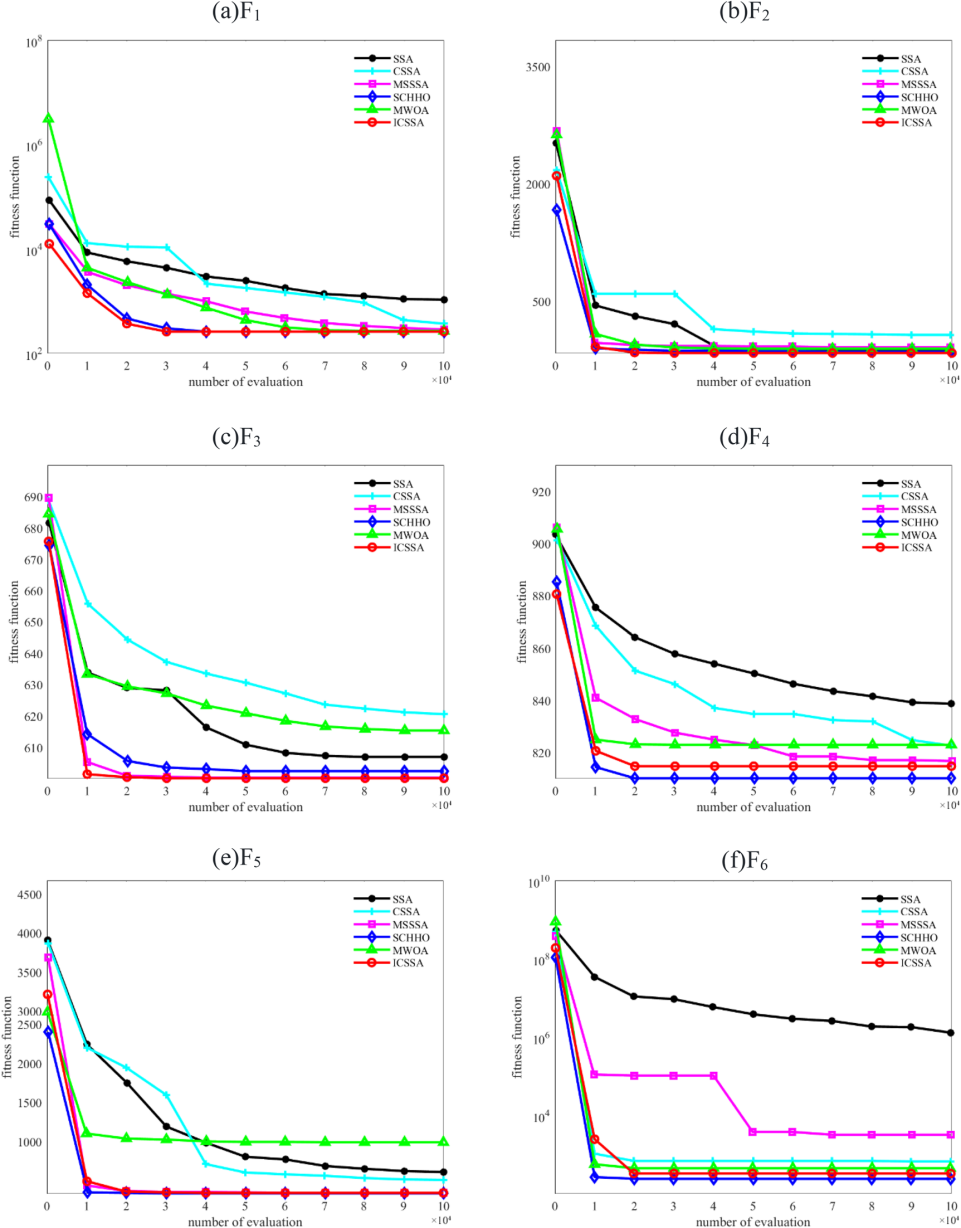

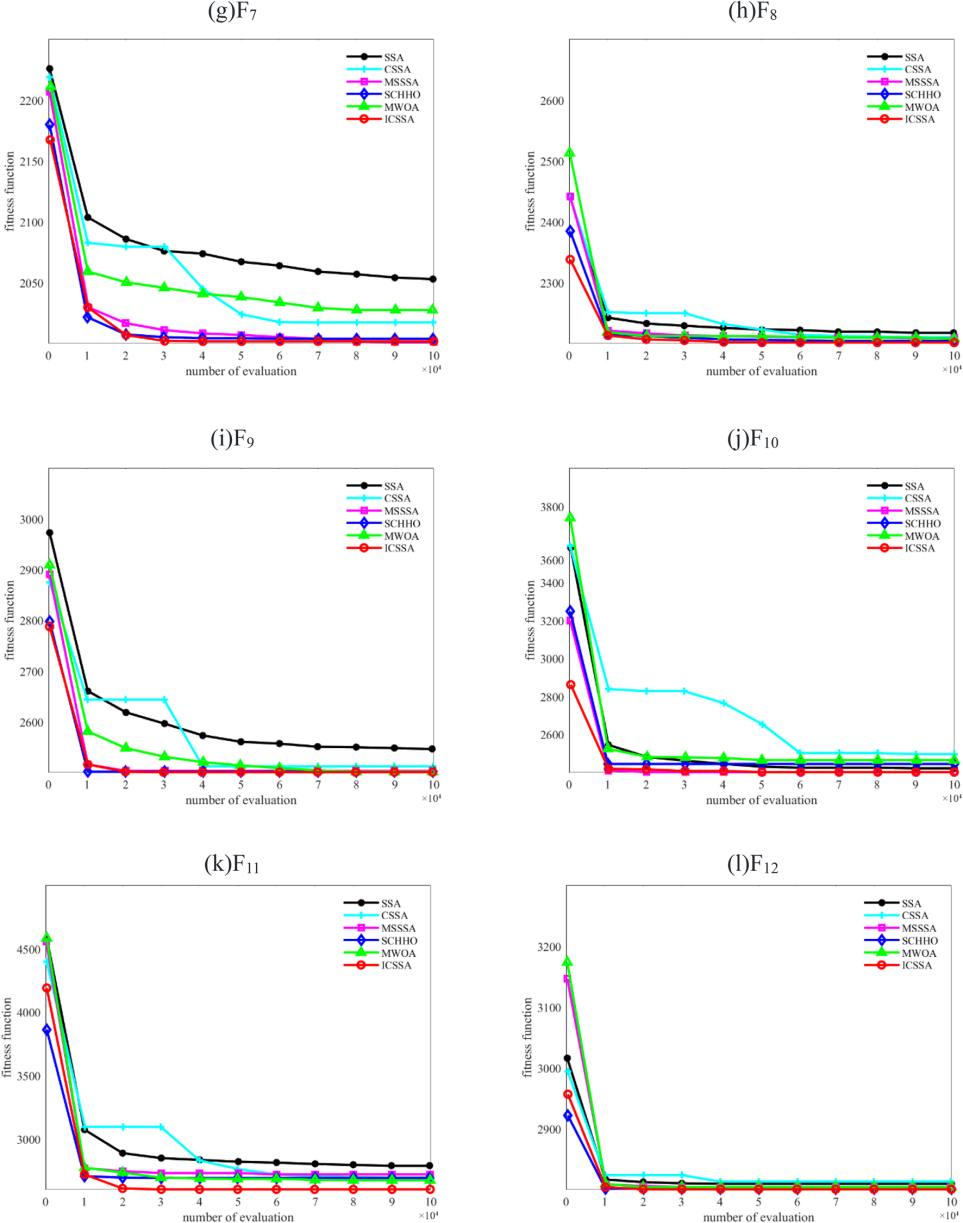
Average convergence curves of CEC2022 test functions.

From Tables 1 and 2, it can be observed that ICSSA demonstrates good performance indicators across various functions. Especially in the case of 10 dimensions, ICSSA can find the optimal values in functions F_1-3_, F_5_, F_7-9_, and F_11_. Although it does not converge to the optimal values in functions F_4_, F_6_, F_10_, and F_12_, it still exhibits better indicators than other algorithms. Similarly, in the case of 20 dimensions, ICSSA also shows superior performance. From Fig. 5, it can be observed that in the four functions where the optimal values were not found, ICSSA demonstrates good convergence speed. Moreover, in functions F_10_ and F_12_, ICSSA exhibits good initial values, indicating a superior initial population distribution. Overall, in the CEC2022 test function set, ICSSA can find the optimal values in most functions, and in the functions where the optimal values are not found, the obtained solutions are still close to the optimal values. Additionally, ICSSA performs well in other indicators, reflecting its generality.

### Ablation experiments

To verify the effectiveness of each improvement point in the algorithm, ablation experiments were conducted. The Sparrow Search Algorithm was enhanced with four different strategies: SSA-1, which incorporates chaotic mapping; SSA-2, which utilizes weight factors; SSA-3, which combines the sine–cosine algorithm; and SSA-4, which introduces lens imaging reverse learning. These four improved versions of the Sparrow Search Algorithm were tested on 10 dimensions using the CEC2022 benchmark functions. The test results are shown in Table 3.

**Table 3:** Comparison of improvement strategies.

Function	Index	SSA-1	SSA-2	SSA-3	SSA-4	ICSSA
F1	Best	3.5429E + 02	2.2217E + 03	3.0722E + 02	2.3229E + 03	**3.0000E + 02**
	Ave	1.3424E + 03	1.0551E + 04	5.4928E + 03	6.2571E + 03	**3.0000E + 02**
	Std	5.8257E + 02	7.1543E + 03	2.0579E + 03	1.6072E + 03	**1.6900E-09**
F2	Best	4.0356E + 02	4.0635E + 02	4.0214E + 02	9.5147E + 02	**4.0000E + 02**
	Ave	4.0837E + 02	4.2437E + 02	4.2014E + 02	1.1735E + 03	**4.0405E + 02**
	Std	1.5424E + 01	2.7685E + 01	4.6323E + 00	3.3942E + 02	**3.6746E + 00**
F3	Best	6.0229E + 02	6.1160E + 02	6.0412E + 02	6.1259E + 02	**6.0000E + 02**
	Ave	6.0544E + 02	6.1465E + 02	6.0824E + 02	6.4173E + 02	**6.0000E + 02**
	Std	4.0615E + 00	9.6642E + 00	2.2633E + 00	1.0581E + 01	**2.0336E + 00**
F4	Best	8.2724E + 02	8.2533E + 02	8.2047E + 02	8.2755E + 02	**8.0246E + 02**
	Ave	8.2343E + 02	8.3524E + 02	8.2678E + 02	8.4780E + 02	**8.2219E + 02**
	Std	9.8923E + 00	1.0907E + 01	7.6914E + 00	6.8727E + 00	**6.1324E + 00**
F5	Best	9.0410E + 02	1.1434E + 03	9.0884E + 02	1.0419E + 03	**9.0000E + 02**
	Ave	9.5218E + 02	1.7728E + 03	9.4028E + 02	1.3018E + 03	**9.0167E + 02**
	Std	8.7078E + 01	5.0331E + 02	2.3923E + 01	1.2049E + 02	**3.5486E + 00**
F6	Best	2.4279E + 03	2.8815E + 03	2.4433E + 03	1.9207E + 04	**1.8239E + 03**
	Ave	3.6312E + 03	4.8163E + 03	1.4940E + 05	6.7250E + 06	**2.6126E + 03**
	Std	4.0445E + 03	2.5169E + 03	1.1565E + 05	7.3220E + 06	**1.2223E + 03**
F7	Best	2.0635E + 03	2.1130E + 03	2.0246E + 03	2.0227E + 03	**2.0000E + 03**
	Ave	2.0314E + 03	2.0516E + 03	2.0384E + 03	2.0972E + 03	**2.0200E + 03**
	Std	1.1527E + 01	3.1244E + 01	4.2073E + 01	1.5715E + 01	**1.0355E + 01**
F8	Best	2.2418E + 03	2.2205E + 03	2.2510E + 03	2.2259E + 03	**2.2000E + 03**
	Ave	2.2604E + 03	2.2442E + 03	2.2891E + 03	2.2352E + 03	**2.2200E + 03**
	Std	5.6423E + 00	4.8993E + 01	3.1507E + 00	5.2825E + 00	**3.1063E + 00**
F9	Best	2.5431E + 03	2.5425E + 03	2.5409E + 03	2.6837E + 03	**2.5317E + 03**
	Ave	2.5523E + 03	2.5489E + 03	2.5412E + 03	2.7215E + 03	**2.5300E + 03**
	Std	7.0824E + 00	1.7176E + 01	9.5027E + 00	3.7909E + 01	**1.0422E-13**
F10	Best	2.5270E + 03	2.5328E + 03	2.5335E + 03	2.5166E + 03	**2.5000E + 03**
	Ave	2.5326E + 03	2.5587E + 03	2.5424E + 03	2.6683E + 03	**2.5003E + 03**
	Std	5.7418E + 01	7.0990E + 01	6.2048E + 01	1.1474E + 02	**4.9157E-02**
F11	Best	2.6110E + 03	2.7542E + 03	2.6733E + 03	2.8912E + 03	**2.6000E + 03**
	Ave	2.6461E + 03	2.8310E + 03	2.7293E + 03	3.4211E + 03	**2.6342E + 03**
	Std	9.7594E + 01	1.8954E + 02	1.9810E + 02	3.2823E + 02	**7.8519E + 01**
F12	Best	2.8650E + 03	2.8615E + 03	2.8644E + 03	2.8853E + 03	**2.8551E + 03**
	Ave	2.8747E + 03	2.8875E + 03	2.8704E + 03	2.9341E + 03	**2.8600E + 03**
	Std	3.0212E + 00	1.9961E + 01	1.2949E + 00	3.8926E + 01	**1.2612E + 00**

Significant values are in [bold].

From Table 3, it can be observed that each improvement strategy has its advantages, but ICSSA consistently achieves the best performance across all indicators. This indicates that ICSSA exhibits a more balanced population initialization, which enhances its global exploration capability. By incorporating the modified sine–cosine algorithm, ICSSA expands the search space and accelerates the exchange of information among individuals in the population. Additionally, the introduction of the lens imaging reverse learning strategy strengthens ICSSA's ability to overcome local optima.

## UAV path planning

### Constrains for UAVs

Unmanned aerial vehicles (UAVs) need to consider their performance and environmental factors to better accomplish their tasks. Therefore, this paper mainly considers constraints such as maximum flight distance, flight altitude, angle constraints, and threat models.

(1) Maximum flight distance.

The large flight distance is determined by the fuel consumption of the UAV and the task, and the maximum flight distance should be given priority when the UAV performs the task. If the longest distance of the UAV is $L_{\max }$, there are $n$ points on a trajectory, and the length of the $i$ section of the trajectory is $L_{i}$, then the length of the entire trajectory is

16 $$L = \sum\limits_{i = 1}^{n - 1} {L_{i} } \,\,\,\,\,\,\,\,\, L \le L_{\max }$$

(2) Flight Altitude.

In the planning space, the maximum flight height of the UAV cannot exceed the planning space, but it cannot be too low, so that it will not crash because of too low flight impact on the ground, so the flight height of the UAV is:

17 $$h_{\min } < h \le h_{\max }$$

(3) Angle Constraints.

The Angle constraint of UAV includes two parts: yaw Angle and pitch Angle. Yaw Angle refers to the turning Angle between two adjacent track nodes of the UAV during flight. Due to the performance limitations of the UAV, the maximum turning Angle in actual flight cannot be unlimited. Therefore, the maximum yaw Angle in the simulation is 60°. The pitch Angle is the Angle between the body axis of the UAV and the horizontal plane. In path planning, the UAV needs to fly to avoid obstacles, but too much pitch Angle will cause the UAV to stall and crash. Therefore, the yaw Angle should meet the following conditions

18 $$\frac{{\left\| {z_{j} - \left. {z_{i}}\right\|} \right.}}{{\sqrt {\left\| {(x_{j} - x_{i} )^{2} + \left. {(y_{j} - y_{i} )^{2} } \right\|} \right.} }} = \tan \gamma$$

(4) Threat Models.

**Figure 6:**
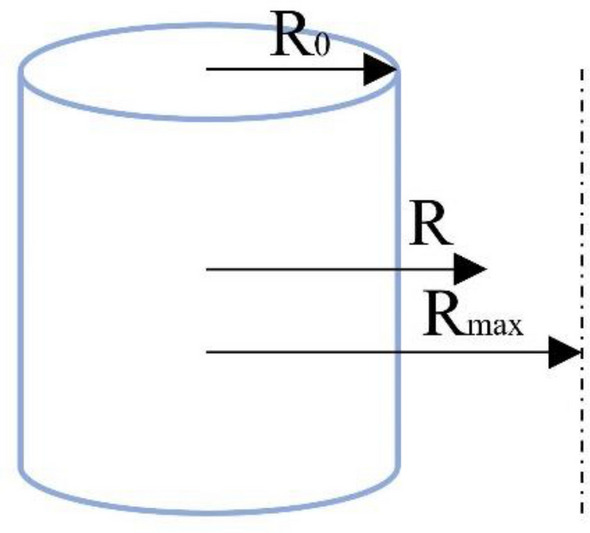
Obstacle model.

There may be other types of obstacles, as well as terrain obstacles when drones perform missions. In this paper, other types of obstacles are normalized and uniformly defined as cylindrical obstacles, as shown in Fig. 6 When the distance between the UAV and the obstacle is greater than $R_{\max }$, it is safe, and when the distance is less than $R_{\max }$ and greater than $R_{0}$, it brakes.

### Cost function

The track cost function is an important index to evaluate the performance of the improved algorithm, and the UAV path planning is to spend the least cost when completing the task. The track cost in this paper is shown in the following equation

19 $$J = \alpha_{1} J_{1} + \alpha_{2} J_{2} + \alpha_{3} J_{3}$$

where $J\arcsin \theta$, $J_{1}$, $J_{2}$ and $J_{3}$ are the total cost, path length cost, height cost, and corner cost respectively; $\alpha_{1}$, $\alpha_{2}$ and $\alpha_{3}$ are the weight coefficients, and their sum is 0.

(1) Path Length Cost.

The path length is an important index of path planning. A shorter path means shorter time and less energy consumption. The path length cost in this paper is:

20 $$J_{1} = \sum\limits_{i = 0}^{n} {\sqrt {(x_{i + 1} - x_{i} )^{2} + (y_{i + 1} + y_{i} )^{2} + (z_{i + 1} - z_{i} )^{2} } }$$

(2) Height Cost.

The stable altitude of the UAV during flight can effectively save energy and ensure safety, in addition, when flying at a low altitude, it can use the ground cover to effectively avoid unknown threats. So the height cost is:

21 $$J_{2} = \sqrt {\frac{1}{n}\sum\limits_{i = 0}^{n - 1} {(z_{i} - \frac{1}{n}\sum\limits_{i = 0}^{n - 1} {z_{i} } )^{2} } }$$

(3) Corner Cost.

The maneuverability and stability of UAVs are limited by the maximum turning Angle between two adjacent waypoints. In addition, frequent turns will reduce flight efficiency, so the Angle cost in this paper is:

22 $$J_{3} = \left\{ \begin{gathered} \sum\limits_{i = 1}^{n} {(\cos \varphi - \cos \theta_{i} )} \, \varphi \ge \theta_{i} \hfill \\ \infty \,\,\,\,\,\,\,\,\,\, \varphi < \theta_{i} \hfill \\ \end{gathered} \right.$$

23 $$\cos \theta = \frac{{a_{i}^{T} \cdot a_{i + 1} }}{{\left| {a_{i} } \right| \cdot \left| {a_{i + 1} } \right|}}$$

where $\varphi$ is the maximum Angle, $\theta$ is the current Angle, and $a_{i}$ is the i-th segment in the entire path.

## Experimental simulation

The experiment was conducted in a 3D mountainous environment with dimensions of 100 × 150 × 5 km, and several obstacles were set up within the environment. The proposed algorithm was compared with CSSA, SSA, and PSO. All algorithms had a population size of 30 and a maximum iteration count of 500. The parameters for PSO were set as ${c}_{1}={c}_{2}=2$ and $\omega =0.65$. For CSSA, the Piecewise chaotic mapping was used. The discoverer percentage for CSSA, SSA, and the proposed algorithm was set to 20%, while the sentinel percentage was set to 15%.

To increase the credibility of the experiments and reduce the interference of accidental events, each algorithm was independently run 30 times, and the path length, running time, and cost were recorded as three performance metrics.

### Scenario one

**Figure 7:**
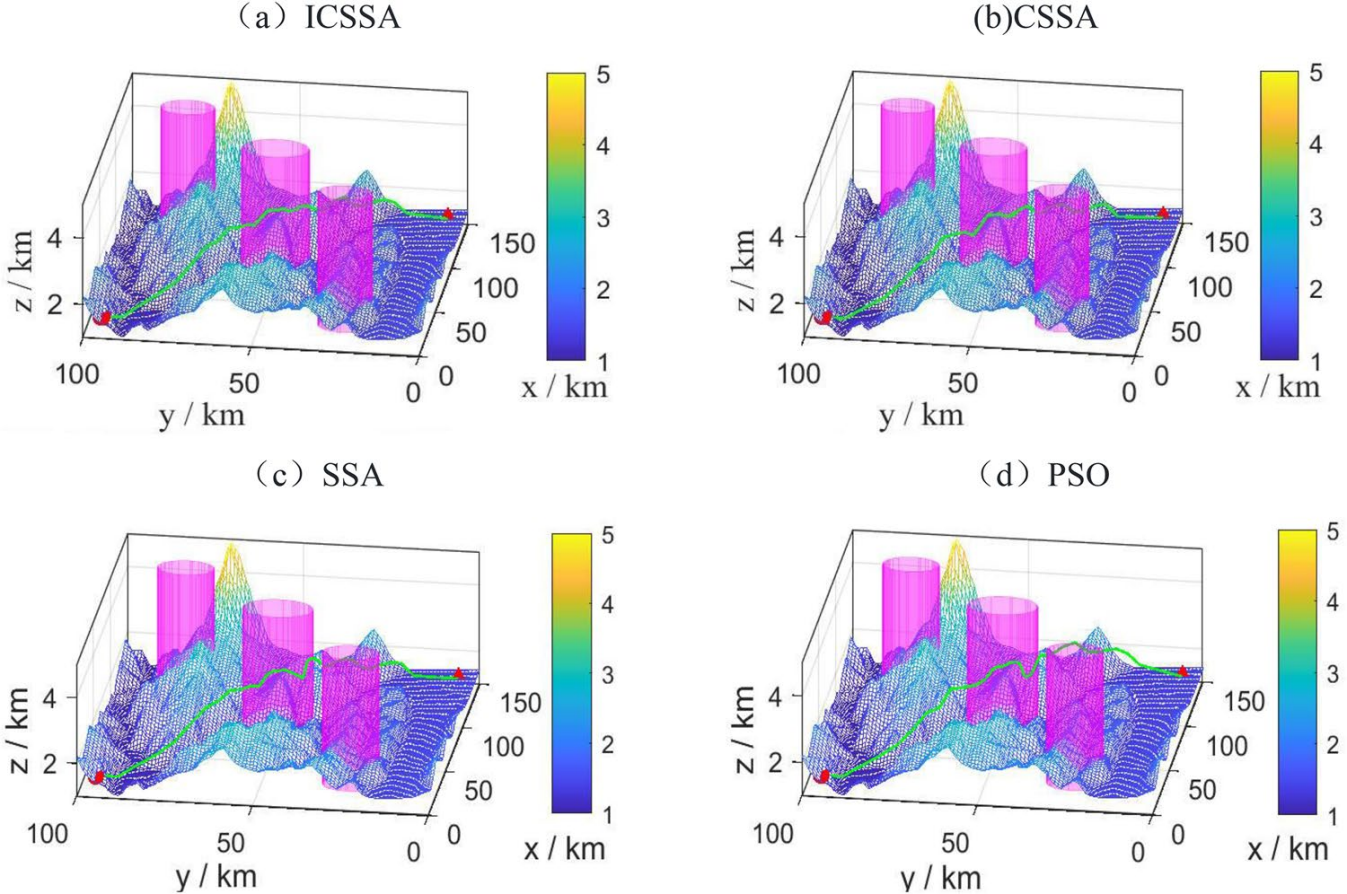
Trajectory diagram of each algorithm.

**Figure 8:**
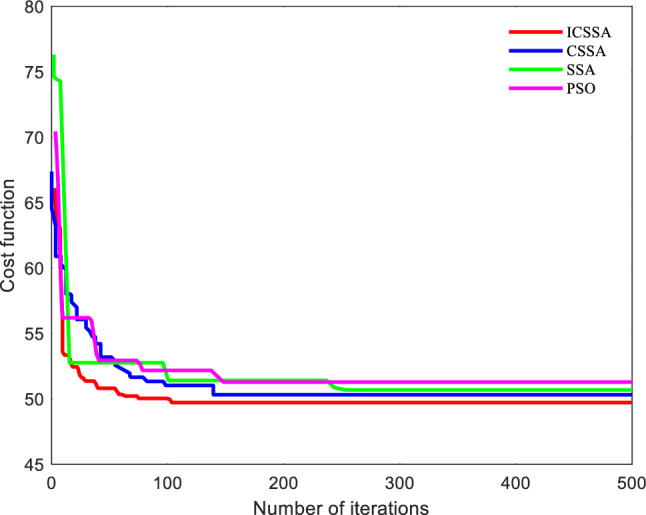
Cost function curves of each algorithm.

To validate the feasibility of the improved algorithm, a relatively simple scenario, referred to as Scene 1, was chosen as the environment map Fig. 7 displays the best route maps for PSO, CSSA, SSA, and the suggested algorithm. Figure 8 displays the cost function curves for the four algorithms.

From Fig. 7, it can be observed that each algorithm can generate a feasible path. By examining the planned paths, it is evident that the path generated in Fig. 7b has less fluctuation compared to Fig. 7c, indicating the effectiveness of introducing chaotic mapping. The path variation in Fig. 7a is smoother and without sudden changes, which is superior to CSSA, SSA, and PSO. This demonstrates the superiority of the proposed algorithm.

From Fig. 8, it can be observed that the proposed algorithm performs better than CSSA and SSA in terms of initial values. Additionally, the traditional SSA algorithm has a slower convergence speed and tends to get stuck in local optima for a longer time in the later iterations. CSSA significantly accelerates the convergence speed and has a stronger ability to escape local optima by introducing chaotic mapping. The proposed algorithm improves convergence accuracy, speed, and search performance significantly by incorporating multiple strategies. Table 4. shows that the planning times for CSSA, SSA, and PSO are 19.9331, 23.4671, and 31.1205, respectively, whereas the suggested algorithm's planning time is 13.3561, a 31.97% decrease from CSSA. In comparison to the 198.6771 km of PSO, the suggested algorithm's path length of 155.4782 km is a significant improvement. The experimental findings show that the suggested approach performs quickly and with good convergence in a simple map context.

**Table 4:** Performance Indicators of Each Algorithm.

Index	Path length /km	Best cost price	Simulation time /s
PSO	198.6771	51.28	31.1205
SSA	165.7981	50.68	23.4671
CSSA	160.4816	50.32	19.9331
ICSSA	**155.4782**	**49.71**	**13.3561**

Significant values are in [bold].

### Scenario two

**Figure 9:**
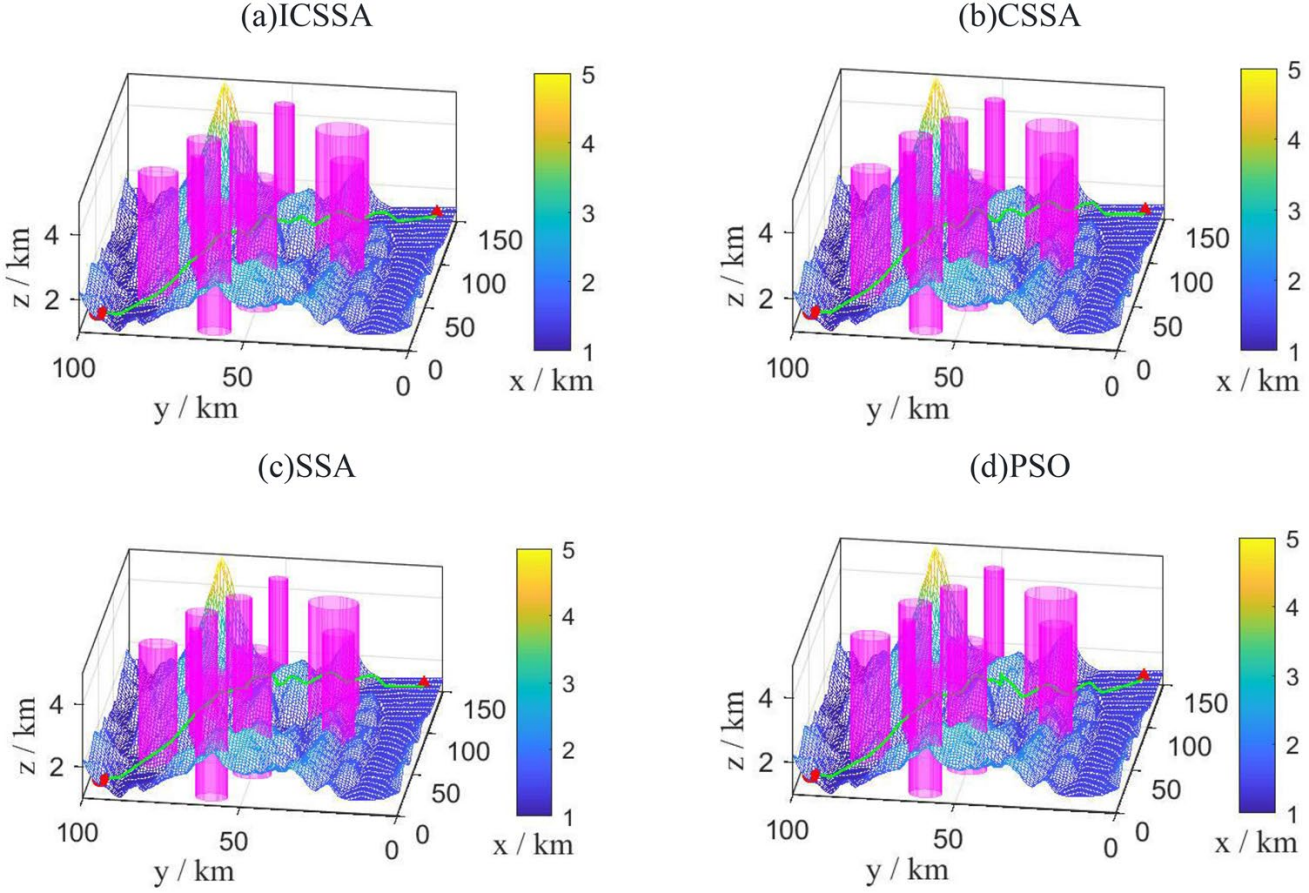
Trajectory diagram of each algorithm.

**Figure 10:**
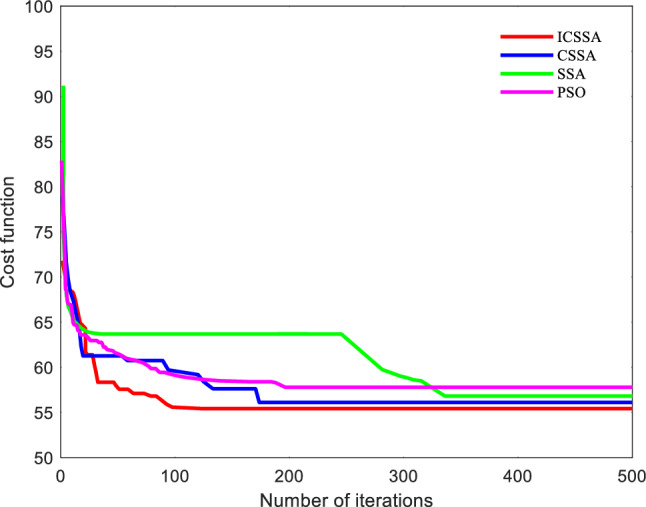
Cost function curve of each algorithm.

A more complex setting was chosen for simulation to verify the suggested algorithm's superiority. The optimal route maps for PSO, GWO, SSA, and the proposed algorithm are shown in Figs. 9 and 10 displays the cost function curves for the four algorithms.

Figure 9 shows that even in a more complicated setting, the suggested technique is still able to produce a reasonably viable and smooth path. Figure 10 illustrates how the suggested approach, which has the lowest cost of the four techniques at 54.71, converges after 120 iterations. This indicates that the proposed algorithm still exhibits good convergence speed in complex environments. According to the planning time in Table 5, SSA, PSO, and GWO have planning times of 33.1698, 46.5887, and 30.7449, respectively, while the proposed algorithm has a planning time of 24.8537, which is slightly improved. The suggested approach has a path length of 165.343 km, which is much longer than the path lengths of the other three algorithms. This shows the proposed algorithm's superiority in complicated situations.

**Table 5:** Performance indicators of each algorithm.

Index	Path length /km	Best cost price	Simulation time /s
PSO	201.5841	57.80	46.5887
SSA	187.9931	56.82	33.1698
CSSA	183.1399	56.11	30.7449
ICSSA	**165.343**	**55.44**	**23.8537**

Significant values are in [bold].

## Conclusion

This paper proposes an improved chaos sparrow search algorithm (ICSSA) for unmanned aerial vehicle (UAV) path planning problems in complex environments. The solution improves population diversity by utilizing the PWLCM chaotic mapping. It balances the algorithm's local and global development capabilities through the use of nonlinear dynamic weights and an improved sine cosine search algorithm. Furthermore, it employs dynamic boundary lens imaging learning to help the algorithm escape local optima. To show the superiority and effectiveness of the suggested method, two sets of experiments on simple and complex maps are used. Experimental findings show that the suggested algorithm converges in simple maps within 100 iterations, and the planning time is reduced by 32.9%, 43.1%, and 57.1%, respectively, when compared to CSSA, SSA, and PSO. In complex maps, the proposed algorithm still exhibits good convergence speed, converging in approximately 120 iterations, and the planning time is improved by 48.8%, 28.1%, and 22.4% compared to CSSA, SSA, and PSO, respectively. The simulation experiments show that the proposed algorithm can achieve fast convergence, reduce planning time, and generate an efficient collision-free path, effectively addressing the issues of UAVs getting stuck in local optima and slow planning time in complex environments. However, there is still uncertainty when introducing chaotic mapping in improving the initial population, and the next step is to ensure that the population distribution is uniform each time it is initialized. Additionally, all the experimental environments for path planning in this paper are known, and mainly involve simulation on mountain maps, with no simulation or real-world experiments conducted on environments such as city maps and indoor maps. Moreover, local path planning was not experimentally explored. The next step will be to consider various environmental conditions and situations where local map information is unknown, to achieve rapid path planning.

## Data Availability

The data that support the findings of this study are available from the corresponding author upon reasonable request.
